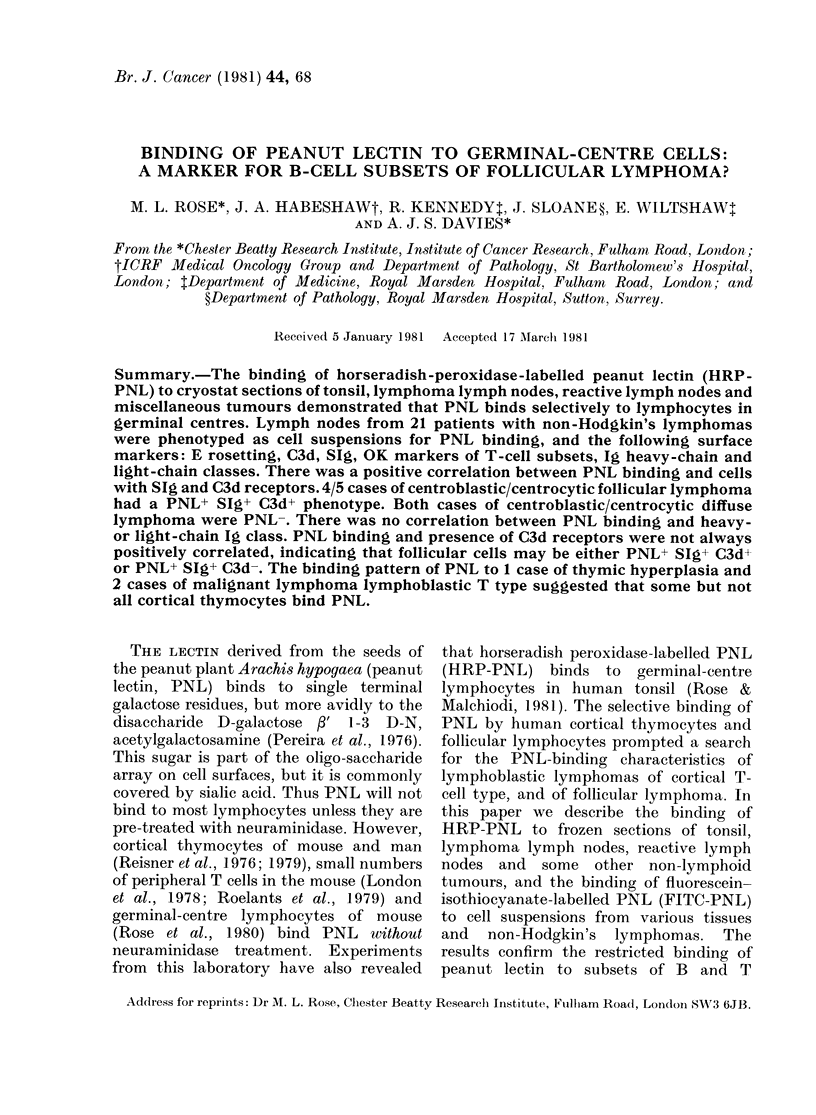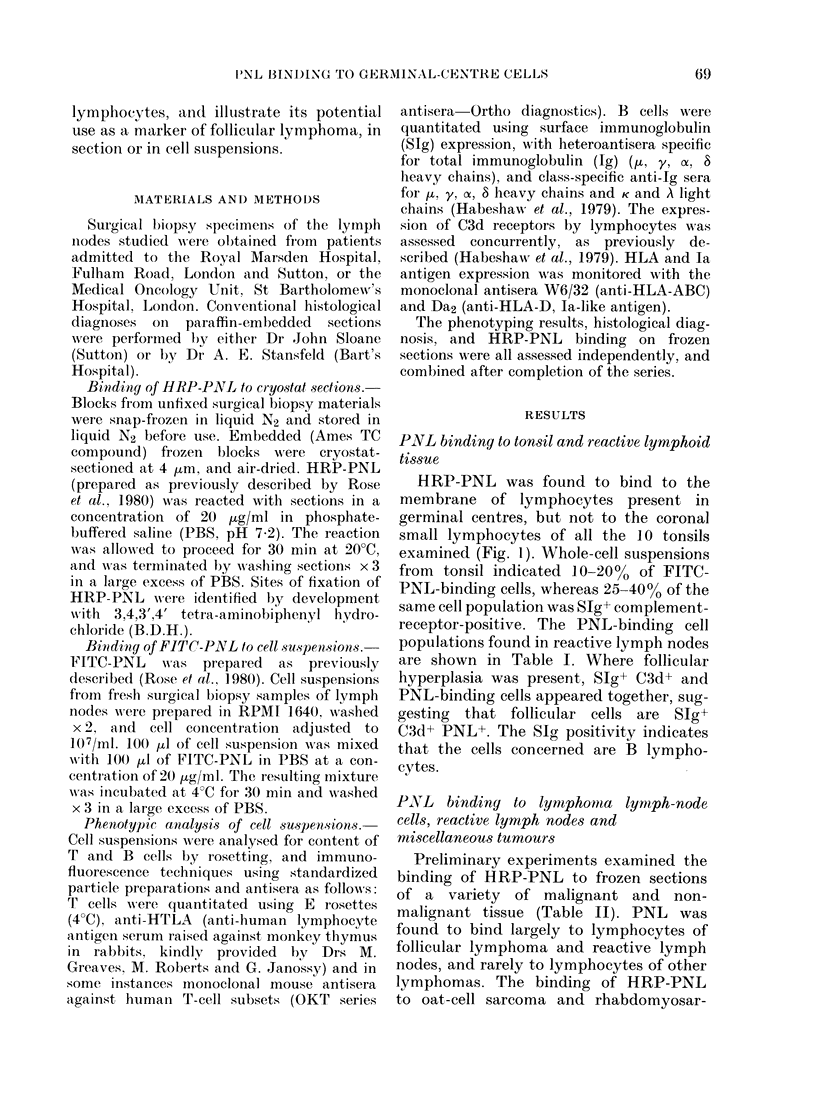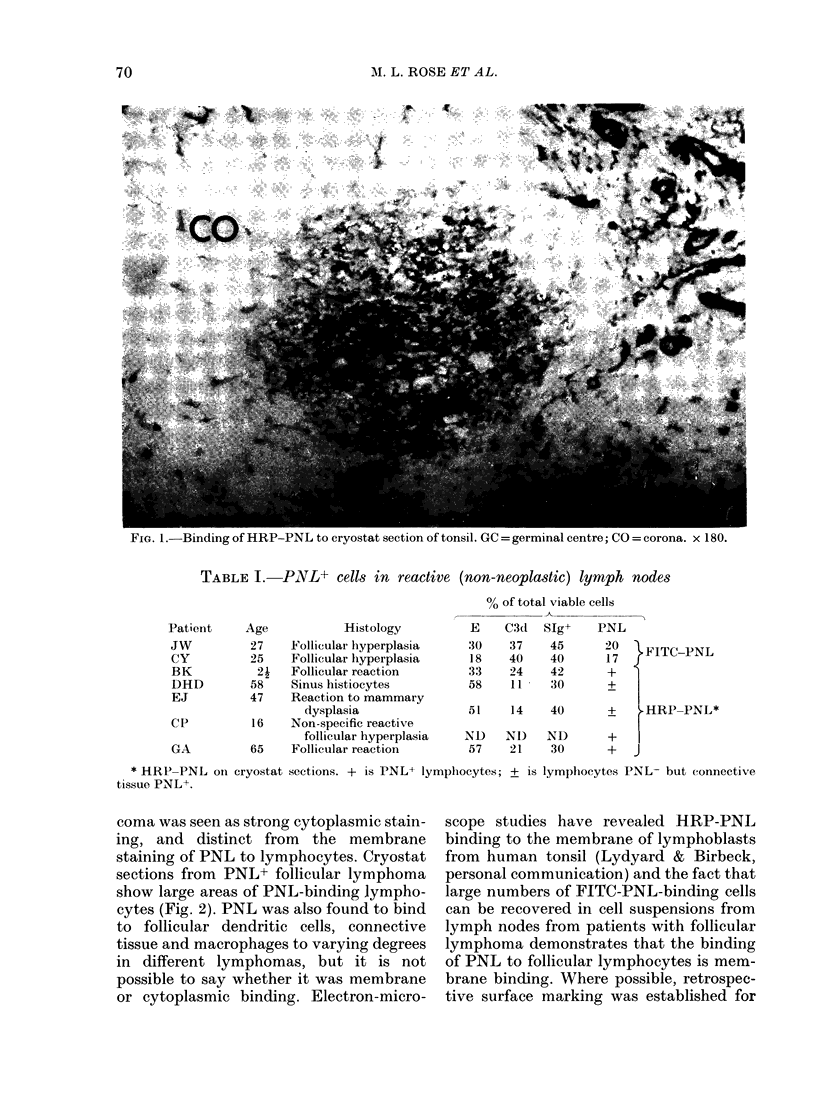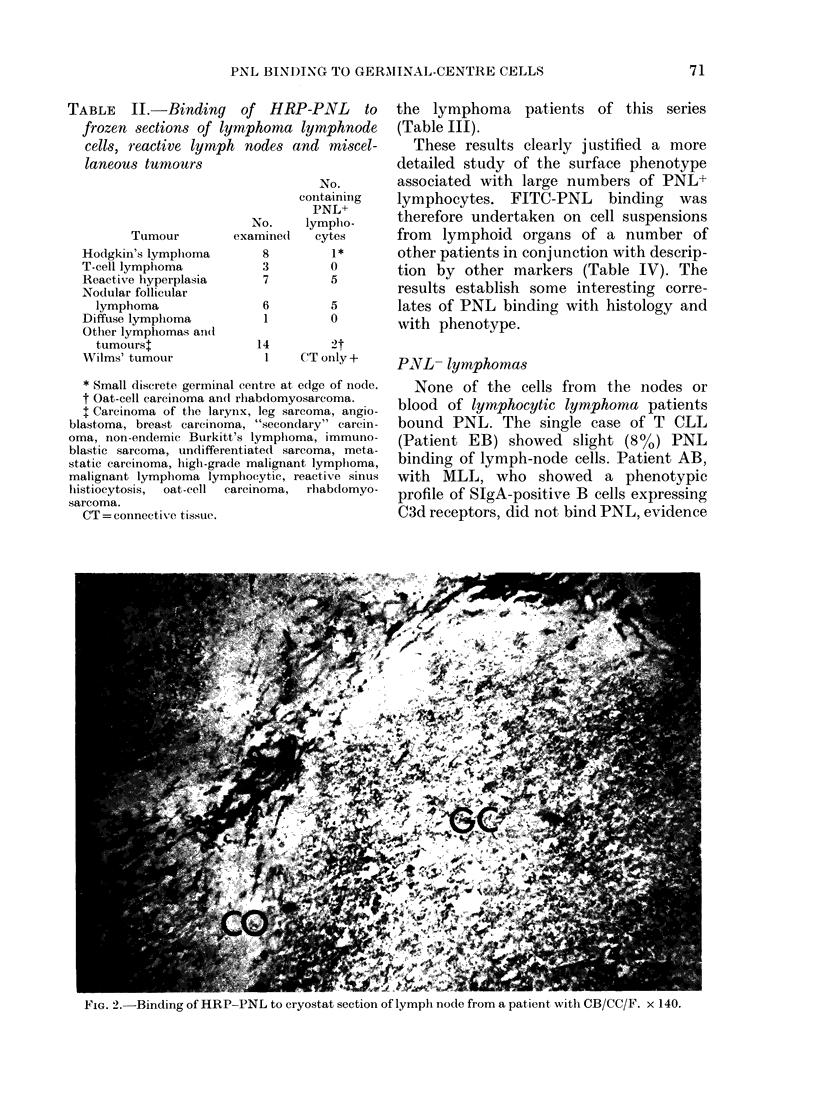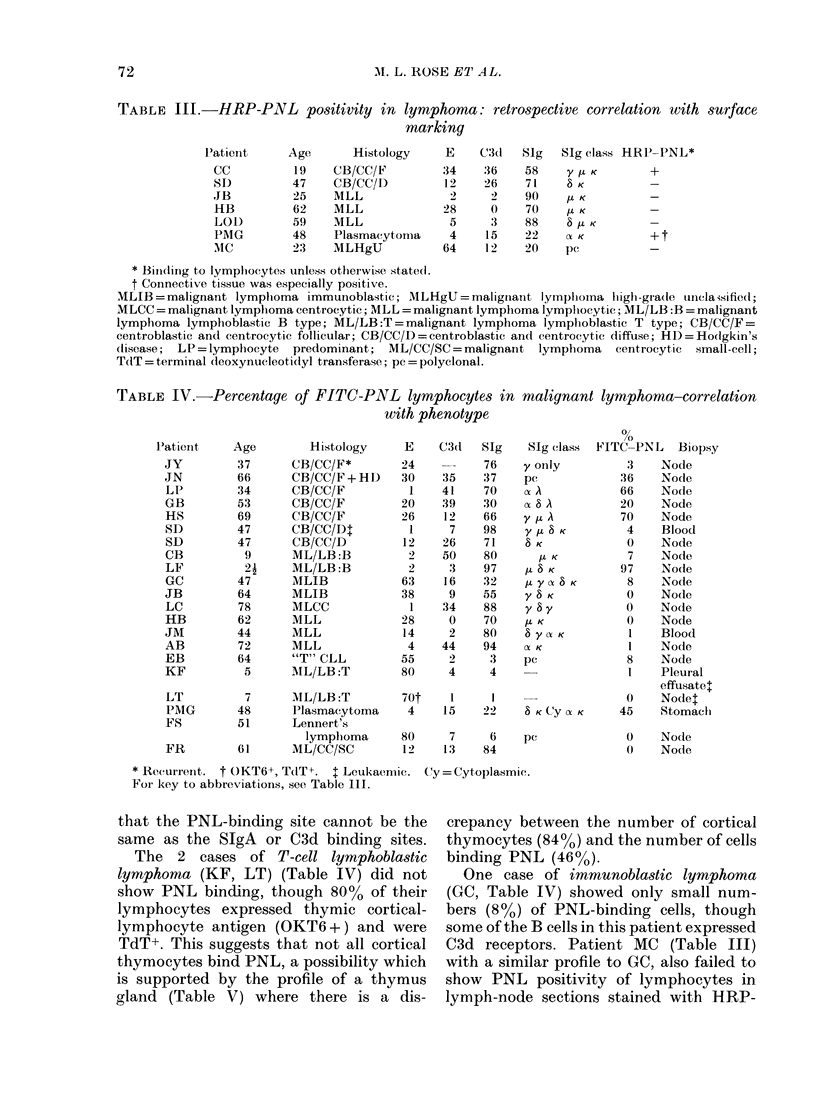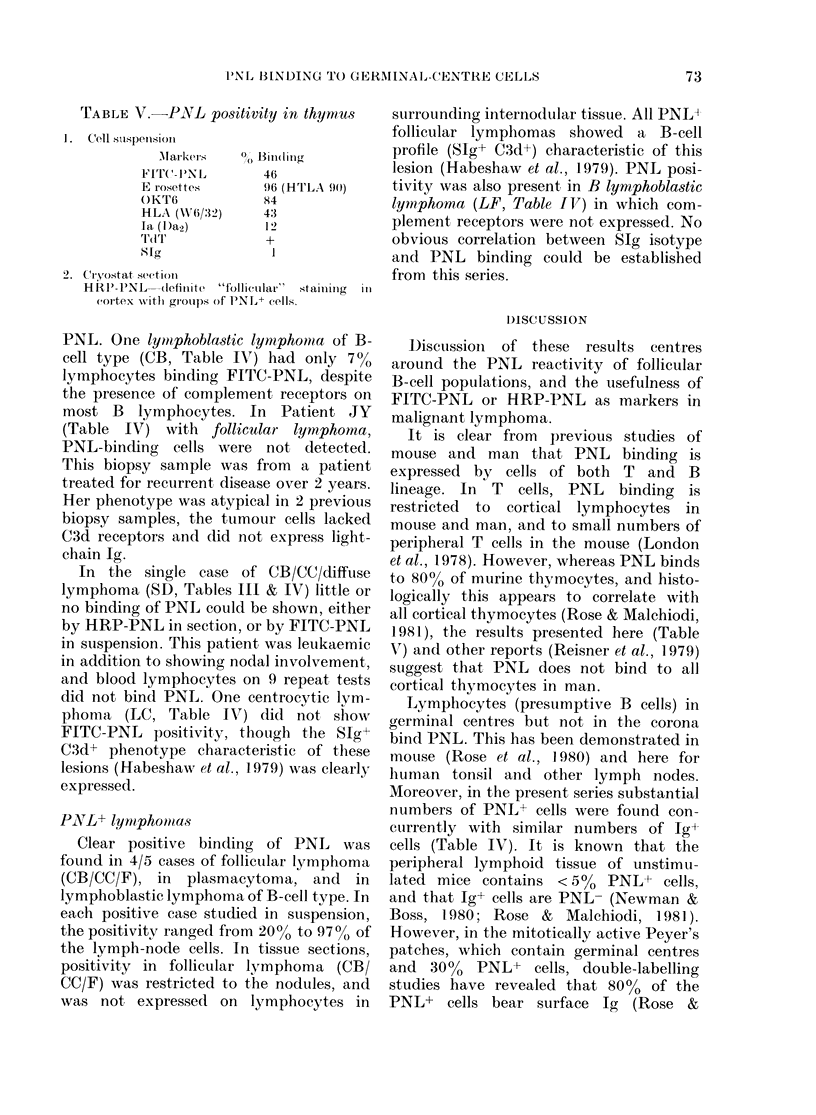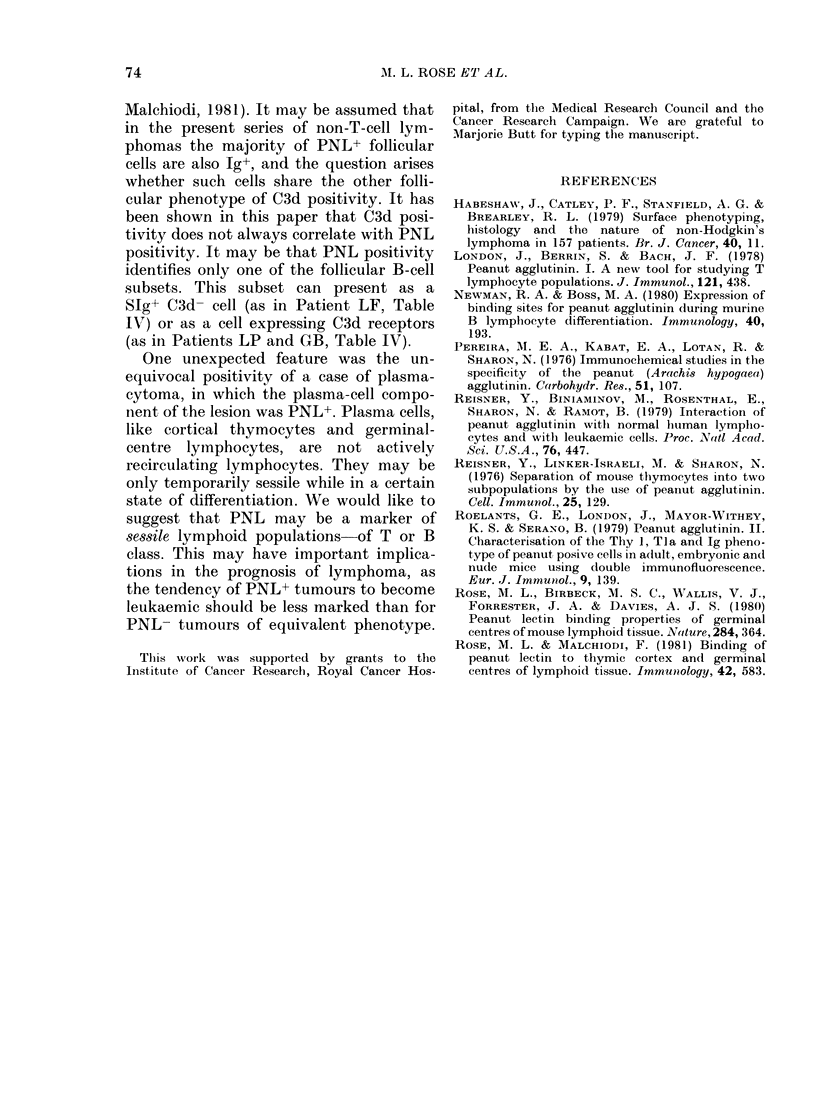# Binding of peanut lectin to germinal-centre cells: a marker for B-cell subsets of follicular lymphoma?

**DOI:** 10.1038/bjc.1981.149

**Published:** 1981-07

**Authors:** M. L. Rose, J. A. Habeshaw, R. Kennedy, J. Sloane, E. Wiltshaw, A. J. Davies

## Abstract

**Images:**


Br. J. Cancer (1981) 44, 68

### BINDING OF PEANUT LECTIN TO GERMINAL-CENTRE CELLS: A MARKER FOR B-CELL SUBSETS OF FOLLICULAR LYMPHOMA?

M. L. ROSE*, J. A. HABESHAW†, R. KENNEDY‡, J. SLOANE§, E. WILTSHAW‡ AND A. J. S. DAVIES*

*From the *Chester Beatty Research Institute, Institute of Cancer Research, Fulham Road, London; †ICRF Medical Oncology Group and Department of Pathology, St Bartholomew's Hospital, London; ‡Department of Medicine, Royal Marsden Hospital, Fulham Road, London; and §Department of Pathology, Royal Marsden Hospital, Sutton, Surrey.*

Received 5 January 1981 Accepted 17 March 1981

**Summary.—The binding of horseradish-peroxidase-labelled peanut lectin (HRP-PNL) to cryostat sections of tonsil, lymphoma lymph nodes, reactive lymph nodes and miscellaneous tumours demonstrated that PNL binds selectively to lymphocytes in germinal centres. Lymph nodes from 21 patients with non-Hodgkin's lymphomas were phenotyped as cell suspensions for PNL binding, and the following surface markers: E rosetting, C3d, SIg, OK markers of T-cell subsets, Ig heavy-chain and light-chain classes. There was a positive correlation between PNL binding and cells with SIg and C3d receptors. 4/5 cases of centroblastic/centrocytic follicular lymphoma had a $PNL^+$ $SIg^+$ $C3d^+$ phenotype. Both cases of centroblastic/centrocytic diffuse lymphoma were $PNL^-$. There was no correlation between PNL binding and heavy- or light-chain Ig class. PNL binding and presence of C3d receptors were not always positively correlated, indicating that follicular cells may be either $PNL^+$ $SIg^+$ $C3d^+$ or $PNL^+$ $SIg^+$ $C3d^-$. The binding pattern of PNL to 1 case of thymic hyperplasia and 2 cases of malignant lymphoma lymphoblastic T type suggested that some but not all cortical thymocytes bind PNL.**

THE LECTIN derived from the seeds of the peanut plant *Arachis hypogaea* (peanut lectin, PNL) binds to single terminal galactose residues, but more avidly to the disaccharide D-galactose β′ 1-3 D-N, acetylgalactosamine (Pereira *et al.*, 1976). This sugar is part of the oligo-saccharide array on cell surfaces, but it is commonly covered by sialic acid. Thus PNL will not bind to most lymphocytes unless they are pre-treated with neuraminidase. However, cortical thymocytes of mouse and man (Reisner *et al.*, 1976; 1979), small numbers of peripheral T cells in the mouse (London *et al.*, 1978; Roelants *et al.*, 1979) and germinal-centre lymphocytes of mouse (Rose *et al.*, 1980) bind PNL *without* neuraminidase treatment. Experiments from this laboratory have also revealed that horseradish peroxidase-labelled PNL (HRP-PNL) binds to germinal-centre lymphocytes in human tonsil (Rose & Malchiodi, 1981). The selective binding of PNL by human cortical thymocytes and follicular lymphocytes prompted a search for the PNL-binding characteristics of lymphoblastic lymphomas of cortical T-cell type, and of follicular lymphoma. In this paper we describe the binding of HRP-PNL to frozen sections of tonsil, lymphoma lymph nodes, reactive lymph nodes and some other non-lymphoid tumours, and the binding of fluorescein-isothiocyanate-labelled PNL (FITC-PNL) to cell suspensions from various tissues and non-Hodgkin's lymphomas. The results confirm the restricted binding of peanut lectin to subsets of B and T

Address for reprints: Dr M. L. Rose, Chester Beatty Research Institute, Fulham Road, London SW3 6JB.

lymphocytes, and illustrate its potential use as a marker of follicular lymphoma, in section or in cell suspensions.

### MATERIALS AND METHODS

Surgical biopsy specimens of the lymph nodes studied were obtained from patients admitted to the Royal Marsden Hospital, Fulham Road, London and Sutton, or the Medical Oncology Unit, St Bartholomew's Hospital, London. Conventional histological diagnoses on paraffin-embedded sections were performed by either Dr John Sloane (Sutton) or by Dr A. E. Stansfeld (Bart's Hospital).

*Binding of HRP-PNL to cryostat sections.*—Blocks from unfixed surgical biopsy materials were snap-frozen in liquid $N_2$ and stored in liquid $N_2$ before use. Embedded (Ames TC compound) frozen blocks were cryostat-sectioned at 4 $\mu$m, and air-dried. HRP-PNL (prepared as previously described by Rose *et al.*, 1980) was reacted with sections in a concentration of 20 $\mu$g/ml in phosphate-buffered saline (PBS, pH 7·2). The reaction was allowed to proceed for 30 min at 20°C, and was terminated by washing sections ×3 in a large excess of PBS. Sites of fixation of HRP-PNL were identified by development with 3,4,3′,4′ tetra-aminobiphenyl hydrochloride (B.D.H.).

*Binding of FITC-PNL to cell suspensions.*—FITC-PNL was prepared as previously described (Rose *et al.*, 1980). Cell suspensions from fresh surgical biopsy samples of lymph nodes were prepared in RPMI 1640, washed ×2, and cell concentration adjusted to $10^7$/ml. 100 $\mu$l of cell suspension was mixed with 100 $\mu$l of FITC-PNL in PBS at a concentration of 20 $\mu$g/ml. The resulting mixture was incubated at 4°C for 30 min and washed ×3 in a large excess of PBS.

*Phenotypic analysis of cell suspensions.*—Cell suspensions were analysed for content of T and B cells by rosetting, and immunofluorescence techniques using standardized particle preparations and antisera as follows: T cells were quantitated using E rosettes (4°C), anti-HTLA (anti-human lymphocyte antigen serum raised against monkey thymus in rabbits, kindly provided by Drs M. Greaves, M. Roberts and G. Janossy) and in some instances monoclonal mouse antisera against human T-cell subsets (OKT series antisera—Ortho diagnostics). B cells were quantitated using surface immunoglobulin (SIg) expression, with heteroantisera specific for total immunoglobulin (Ig) ($\mu$, $\gamma$, $\alpha$, $\delta$ heavy chains), and class-specific anti-Ig sera for $\mu$, $\gamma$, $\alpha$, $\delta$ heavy chains and $\kappa$ and $\lambda$ light chains (Habeshaw *et al.*, 1979). The expression of C3d receptors by lymphocytes was assessed concurrently, as previously described (Habeshaw *et al.*, 1979). HLA and Ia antigen expression was monitored with the monoclonal antisera W6/32 (anti-HLA-ABC) and $Da_2$ (anti-HLA-D, Ia-like antigen).

The phenotyping results, histological diagnosis, and HRP-PNL binding on frozen sections were all assessed independently, and combined after completion of the series.

### RESULTS

### *PNL binding to tonsil and reactive lymphoid tissue*

HRP-PNL was found to bind to the membrane of lymphocytes present in germinal centres, but not to the coronal small lymphocytes of all the 10 tonsils examined (Fig. 1). Whole-cell suspensions from tonsil indicated 10–20% of FITC-PNL-binding cells, whereas 25–40% of the same cell population was $SIg^+$ complement-receptor-positive. The PNL-binding cell populations found in reactive lymph nodes are shown in Table I. Where follicular hyperplasia was present, $SIg^+$ $C3d^+$ and PNL-binding cells appeared together, suggesting that follicular cells are $SIg^+$ $C3d^+$ $PNL^+$. The SIg positivity indicates that the cells concerned are B lymphocytes.

### *PNL binding to lymphoma lymph-node cells, reactive lymph nodes and miscellaneous tumours*

Preliminary experiments examined the binding of HRP-PNL to frozen sections of a variety of malignant and non-malignant tissue (Table II). PNL was found to bind largely to lymphocytes of follicular lymphoma and reactive lymph nodes, and rarely to lymphocytes of other lymphomas. The binding of HRP-PNL to oat-cell sarcoma and rhabdomyosar-

FIG. 1.—Binding of HRP–PNL to cryostat section of tonsil. GC = germinal centre; CO = corona. × 180.

TABLE I.—*$PNL^+$ cells in reactive (non-neoplastic) lymph nodes*

| Patient | Age | Histology | E | C3d | $SIg^+$ | PNL | |
|---|---|---|---|---|---|---|---|
| | | | % of total viable cells | | | | |
| JW | 27 | Follicular hyperplasia | 30 | 37 | 45 | 20 | FITC–PNL |
| CY | 25 | Follicular hyperplasia | 18 | 40 | 40 | 17 | FITC–PNL |
| BK | 2½ | Follicular reaction | 33 | 24 | 42 | + | HRP–PNL* |
| DHD | 58 | Sinus histiocytes | 58 | 11 | 30 | ± | HRP–PNL* |
| EJ | 47 | Reaction to mammary dysplasia | 51 | 14 | 40 | ± | HRP–PNL* |
| CP | 16 | Non-specific reactive follicular hyperplasia | ND | ND | ND | + | HRP–PNL* |
| GA | 65 | Follicular reaction | 57 | 21 | 30 | + | HRP–PNL* |

* HRP–PNL on cryostat sections. + is $PNL^+$ lymphocytes; ± is lymphocytes $PNL^-$ but connective tissue $PNL^+$.

coma was seen as strong cytoplasmic staining, and distinct from the membrane staining of PNL to lymphocytes. Cryostat sections from $PNL^+$ follicular lymphoma show large areas of PNL-binding lymphocytes (Fig. 2). PNL was also found to bind to follicular dendritic cells, connective tissue and macrophages to varying degrees in different lymphomas, but it is not possible to say whether it was membrane or cytoplasmic binding. Electron-microscope studies have revealed HRP-PNL binding to the membrane of lymphoblasts from human tonsil (Lydyard & Birbeck, personal communication) and the fact that large numbers of FITC-PNL-binding cells can be recovered in cell suspensions from lymph nodes from patients with follicular lymphoma demonstrates that the binding of PNL to follicular lymphocytes is membrane binding. Where possible, retrospective surface marking was established for

TABLE II.—*Binding of HRP-PNL to frozen sections of lymphoma lymphnode cells, reactive lymph nodes and miscellaneous tumours*

| Tumour | No. examined | No. containing PNL+ lymphocytes |
|---|---|---|
| Hodgkin's lymphoma | 8 | 1* |
| T-cell lymphoma | 3 | 0 |
| Reactive hyperplasia | 7 | 5 |
| Nodular follicular lymphoma | 6 | 5 |
| Diffuse lymphoma | 1 | 0 |
| Other lymphomas and tumours‡ | 14 | 2† |
| Wilms' tumour | 1 | CT only + |

* Small discrete germinal centre at edge of node.
† Oat-cell carcinoma and rhabdomyosarcoma.
‡ Carcinoma of the larynx, leg sarcoma, angioblastoma, breast carcinoma, "secondary" carcinoma, non-endemic Burkitt's lymphoma, immunoblastic sarcoma, undifferentiated sarcoma, metastatic carcinoma, high-grade malignant lymphoma, malignant lymphoma lymphocytic, reactive sinus histiocytosis, oat-cell carcinoma, rhabdomyosarcoma.
CT = connective tissue.

the lymphoma patients of this series (Table III).

These results clearly justified a more detailed study of the surface phenotype associated with large numbers of PNL+ lymphocytes. FITC-PNL binding was therefore undertaken on cell suspensions from lymphoid organs of a number of other patients in conjunction with description by other markers (Table IV). The results establish some interesting correlates of PNL binding with histology and with phenotype.

### *PNL⁻ lymphomas*

None of the cells from the nodes or blood of *lymphocytic lymphoma* patients bound PNL. The single case of T CLL (Patient EB) showed slight (8%) PNL binding of lymph-node cells. Patient AB, with MLL, who showed a phenotypic profile of SIgA-positive B cells expressing C3d receptors, did not bind PNL, evidence

FIG. 2.—Binding of HRP–PNL to cryostat section of lymph node from a patient with CB/CC/F. × 140.

TABLE III.—*HRP-PNL positivity in lymphoma: retrospective correlation with surface marking*

| Patient | Age | Histology | E | C3d | SIg | SIg class | HRP-PNL* |
|---|---|---|---|---|---|---|---|
| CC | 19 | CB/CC/F | 34 | 36 | 58 | γ μ κ | + |
| SD | 47 | CB/CC/D | 12 | 26 | 71 | δ κ | – |
| JB | 25 | MLL | 2 | 2 | 90 | μ κ | – |
| HB | 62 | MLL | 28 | 0 | 70 | μ κ | – |
| LOD | 59 | MLL | 5 | 3 | 88 | δ μ κ | – |
| PMG | 48 | Plasmacytoma | 4 | 15 | 22 | α κ | +† |
| MC | 23 | MLHgU | 64 | 12 | 20 | pc | – |

* Binding to lymphocytes unless otherwise stated.
† Connective tissue was especially positive.
MLIB = malignant lymphoma immunoblastic; MLHgU = malignant lymphoma high-grade unclassified; MLCC = malignant lymphoma centrocytic; MLL = malignant lymphoma lymphocytic; ML/LB:B = malignant lymphoma lymphoblastic B type; ML/LB:T = malignant lymphoma lymphoblastic T type; CB/CC/F = centroblastic and centrocytic follicular; CB/CC/D = centroblastic and centrocytic diffuse; HD = Hodgkin's disease; LP = lymphocyte predominant; ML/CC/SC = malignant lymphoma centrocytic small-cell; TdT = terminal deoxynucleotidyl transferase; pc = polyclonal.

TABLE IV.—*Percentage of FITC-PNL lymphocytes in malignant lymphoma–correlation with phenotype*

| Patient | Age | Histology | E | C3d | SIg | SIg class | % FITC–PNL | Biopsy |
|---|---|---|---|---|---|---|---|---|
| JY | 37 | CB/CC/F* | 24 | — | 76 | γ only | 3 | Node |
| JN | 66 | CB/CC/F+HD | 30 | 35 | 37 | pc | 36 | Node |
| LP | 34 | CB/CC/F | 1 | 41 | 70 | α λ | 66 | Node |
| GB | 53 | CB/CC/F | 20 | 39 | 30 | α δ λ | 20 | Node |
| HS | 69 | CB/CC/F | 26 | 12 | 66 | γ μ λ | 70 | Node |
| SD | 47 | CB/CC/D‡ | 1 | 7 | 98 | γ μ δ κ | 4 | Blood |
| SD | 47 | CB/CC/D | 12 | 26 | 71 | δ κ | 0 | Node |
| CB | 9 | ML/LB:B | 2 | 50 | 80 | μ κ | 7 | Node |
| LF | 2½ | ML/LB:B | 2 | 3 | 97 | μ δ κ | 97 | Node |
| GC | 47 | MLIB | 63 | 16 | 32 | μ γ α δ κ | 8 | Node |
| JB | 64 | MLIB | 38 | 9 | 55 | γ δ κ | 0 | Node |
| LC | 78 | MLCC | 1 | 34 | 88 | γ δ γ | 0 | Node |
| HB | 62 | MLL | 28 | 0 | 70 | μ κ | 0 | Node |
| JM | 44 | MLL | 14 | 2 | 80 | δ γ α κ | 1 | Blood |
| AB | 72 | MLL | 4 | 44 | 94 | α κ | 1 | Node |
| EB | 64 | "T" CLL | 55 | 2 | 3 | pc | 8 | Node |
| KF | 5 | ML/LB:T | 80 | 4 | 4 | — | 1 | Pleural effusate‡ |
| LT | 7 | ML/LB:T | 70† | 1 | 1 | — | 0 | Node‡ |
| PMG | 48 | Plasmacytoma | 4 | 15 | 22 | δ κ Cy α κ | 45 | Stomach |
| FS | 51 | Lennert's lymphoma | 80 | 7 | 6 | pc | 0 | Node |
| FR | 61 | ML/CC/SC | 12 | 13 | 84 | | 0 | Node |

* Recurrent. † OKT6+, TdT+. ‡ Leukaemic. Cy = Cytoplasmic.

For key to abbreviations, see Table III.

that the PNL-binding site cannot be the same as the SIgA or C3d binding sites.

The 2 cases of *T-cell lymphoblastic lymphoma* (KF, LT) (Table IV) did not show PNL binding, though 80% of their lymphocytes expressed thymic cortical-lymphocyte antigen (OKT6+) and were TdT+. This suggests that not all cortical thymocytes bind PNL, a possibility which is supported by the profile of a thymus gland (Table V) where there is a discrepancy between the number of cortical thymocytes (84%) and the number of cells binding PNL (46%).

One case of *immunoblastic lymphoma* (GC, Table IV) showed only small numbers (8%) of PNL-binding cells, though some of the B cells in this patient expressed C3d receptors. Patient MC (Table III) with a similar profile to GC, also failed to show PNL positivity of lymphocytes in lymph-node sections stained with HRP-

TABLE V.—*PNL positivity in thymus*

1. Cell suspension

| Markers | % Binding |
|---|---|
| FITC-PNL | 46 |
| E rosettes | 96 (HTLA 90) |
| OKT6 | 84 |
| HLA (W6/32) | 43 |
| Ia ($Da_2$) | 12 |
| TdT | + |
| SIg | 1 |

2. Cryostat section

HRP-PNL—definite "follicular" staining in cortex with groups of $PNL^+$ cells.

PNL. One *lymphoblastic lymphoma* of B-cell type (CB, Table IV) had only 7% lymphocytes binding FITC-PNL, despite the presence of complement receptors on most B lymphocytes. In Patient JY (Table IV) with *follicular lymphoma*, PNL-binding cells were not detected. This biopsy sample was from a patient treated for recurrent disease over 2 years. Her phenotype was atypical in 2 previous biopsy samples, the tumour cells lacked C3d receptors and did not express light-chain Ig.

In the single case of CB/CC/diffuse lymphoma (SD, Tables III & IV) little or no binding of PNL could be shown, either by HRP-PNL in section, or by FITC-PNL in suspension. This patient was leukaemic in addition to showing nodal involvement, and blood lymphocytes on 9 repeat tests did not bind PNL. One centrocytic lymphoma (LC, Table IV) did not show FITC-PNL positivity, though the $SIg^+$ $C3d^+$ phenotype characteristic of these lesions (Habeshaw *et al.*, 1979) was clearly expressed.

### *$PNL^+$ lymphomas*

Clear positive binding of PNL was found in 4/5 cases of follicular lymphoma (CB/CC/F), in plasmacytoma, and in lymphoblastic lymphoma of B-cell type. In each positive case studied in suspension, the positivity ranged from 20% to 97% of the lymph-node cells. In tissue sections, positivity in follicular lymphoma (CB/CC/F) was restricted to the nodules, and was not expressed on lymphocytes in surrounding internodular tissue. All $PNL^+$ follicular lymphomas showed a B-cell profile ($SIg^+$ $C3d^+$) characteristic of this lesion (Habeshaw *et al.*, 1979). PNL positivity was also present in *B lymphoblastic lymphoma* (*LF*, *Table IV*) in which complement receptors were not expressed. No obvious correlation between SIg isotype and PNL binding could be established from this series.

### DISCUSSION

Discussion of these results centres around the PNL reactivity of follicular B-cell populations, and the usefulness of FITC-PNL or HRP-PNL as markers in malignant lymphoma.

It is clear from previous studies of mouse and man that PNL binding is expressed by cells of both T and B lineage. In T cells, PNL binding is restricted to cortical lymphocytes in mouse and man, and to small numbers of peripheral T cells in the mouse (London *et al.*, 1978). However, whereas PNL binds to 80% of murine thymocytes, and histologically this appears to correlate with all cortical thymocytes (Rose & Malchiodi, 1981), the results presented here (Table V) and other reports (Reisner *et al.*, 1979) suggest that PNL does not bind to all cortical thymocytes in man.

Lymphocytes (presumptive B cells) in germinal centres but not in the corona bind PNL. This has been demonstrated in mouse (Rose *et al.*, 1980) and here for human tonsil and other lymph nodes. Moreover, in the present series substantial numbers of $PNL^+$ cells were found concurrently with similar numbers of $Ig^+$ cells (Table IV). It is known that the peripheral lymphoid tissue of unstimulated mice contains $<5\%$ $PNL^+$ cells, and that $Ig^+$ cells are $PNL^-$ (Newman & Boss, 1980; Rose & Malchiodi, 1981). However, in the mitotically active Peyer's patches, which contain germinal centres and 30% $PNL^+$ cells, double-labelling studies have revealed that 80% of the $PNL^+$ cells bear surface Ig (Rose &

Malchiodi, 1981). It may be assumed that in the present series of non-T-cell lymphomas the majority of $PNL^+$ follicular cells are also $Ig^+$, and the question arises whether such cells share the other follicular phenotype of C3d positivity. It has been shown in this paper that C3d positivity does not always correlate with PNL positivity. It may be that PNL positivity identifies only one of the follicular B-cell subsets. This subset can present as a $SIg^+$ $C3d^-$ cell (as in Patient LF, Table IV) or as a cell expressing C3d receptors (as in Patients LP and GB, Table IV).

One unexpected feature was the unequivocal positivity of a case of plasmacytoma, in which the plasma-cell component of the lesion was $PNL^+$. Plasma cells, like cortical thymocytes and germinal-centre lymphocytes, are not actively recirculating lymphocytes. They may be only temporarily sessile while in a certain state of differentiation. We would like to suggest that PNL may be a marker of *sessile* lymphoid populations—of T or B class. This may have important implications in the prognosis of lymphoma, as the tendency of $PNL^+$ tumours to become leukaemic should be less marked than for $PNL^-$ tumours of equivalent phenotype.

This work was supported by grants to the Institute of Cancer Research, Royal Cancer Hospital, from the Medical Research Council and the Cancer Research Campaign. We are grateful to Marjorie Butt for typing the manuscript.

REFERENCES

HABESHAW, J., CATLEY, P. F., STANFIELD, A. G. & BREARLEY, R. L. (1979) Surface phenotyping, histology and the nature of non-Hodgkin's lymphoma in 157 patients. *Br. J. Cancer*, **40,** 11.

LONDON, J., BERRIN, S. & BACH, J. F. (1978) Peanut agglutinin. I. A new tool for studying T lymphocyte populations. *J. Immunol.*, **121,** 438.

NEWMAN, R. A. & BOSS, M. A. (1980) Expression of binding sites for peanut agglutinin during murine B lymphocyte differentiation. *Immunology,* **40,** 193.

PEREIRA, M. E. A., KABAT, E. A., LOTAN, R. & SHARON, N. (1976) Immunochemical studies in the specificity of the peanut (*Arachis hypogaea*) agglutinin. *Carbohydr. Res.*, **51,** 107.

REISNER, Y., BINIAMINOV, M., ROSENTHAL, E., SHARON, N. & RAMOT, B. (1979) Interaction of peanut agglutinin with normal human lymphocytes and with leukaemic cells. *Proc. Natl Acad. Sci. U.S.A.*, **76,** 447.

REISNER, Y., LINKER-ISRAELI, M. & SHARON, N. (1976) Separation of mouse thymocytes into two subpopulations by the use of peanut agglutinin. *Cell. Immunol.*, **25,** 129.

ROELANTS, G. E., LONDON, J., MAYOR-WITHEY, K. S. & SERANO, B. (1979) Peanut agglutinin. II. Characterisation of the Thy 1, Tla and Ig phenotype of peanut posive cells in adult, embryonic and nude mice using double immunofluorescence. *Eur. J. Immunol.*, **9,** 139.

ROSE, M. L., BIRBECK, M. S. C., WALLIS, V. J., FORRESTER, J. A. & DAVIES, A. J. S. (1980) Peanut lectin binding properties of germinal centres of mouse lymphoid tissue. *Nature*, **284,** 364.

ROSE, M. L. & MALCHIODI, F. (1981) Binding of peanut lectin to thymic cortex and germinal centres of lymphoid tissue. *Immunology*, **42,** 583.